# Associations between metabolic syndrome components and markers of inflammation in Welsh school children

**DOI:** 10.1007/s00431-017-3065-y

**Published:** 2017-12-22

**Authors:** Non Eleri Thomas, David A. Rowe, Elaine M. Murtagh, Jeffrey W. Stephens, Rhys Williams

**Affiliations:** 1 College of Human and Health Sciences, Swansea University, Swansea, UK; 2 Physical Activity for Health Research Group, University of Strathclyde, Glasgow, UK; 3 Department of Arts Education and Physical Education, Mary Immaculate College, University of Limerick, Limerick, Ireland; 4 Diabetes Research Group, Institute of Life Science, Swansea University, Swansea, UK; 5 The Medical School, Swansea University, Swansea, UK

**Keywords:** Multivariate statistics, Adolescents, Waist circumference, C-reactive protein, Fibrinogen

## Abstract

We investigated the multivariate dimensionality and strength of the relationship between metabolic syndrome (MetS) and inflammation in children. Caucasian school children (*N* = 229; 12–14 years) from Wales were tested on several health indicators including measures of body composition, inflammation, fasting glucose regulation, blood pressure, and lipids. The multivariate association between MetS and inflammation was investigated via canonical correlation analysis. Data were corrected for non-normality by log transformation, and sex-specific *z*-scores computed for variables where there was a significant sex difference. Structure r’s were interpreted to determine the dimensions of MetS and inflammation responsible for significant canonical variates. The overall multivariate association between MetS and inflammation was significant (Wilks’ Lambda = 0.54, *p* < 0.001). The relationship was explained primarily by the waist circumference dimension of MetS (CC = 0.87) and inflammatory markers of fibrinogen (CC = 0.52) and C-reactive protein (CC = 0.50). The pattern of results was similar regardless of whether variables were adjusted for sex differences.

*Conclusion*: Central adiposity is the strongest predictor of the inflammatory aspect of cardiovascular disease risk in Caucasian adolescents. Future research into MetS and cardiometabolic risk should consider multivariate statistical approaches, in order to identify the separate contributions of each dimension in interrelationships and to identify which dimensions are influenced by preventive interventions.

**Table:** 

**What is Known:** • *Metabolic syndrome (MetS) is associated with increased risk of cardiovascular disease (CVD) and type 2 diabetes. Markers of inflammation are also potential predictors of later development of CVD and type 2 diabetes.* • *The contribution of individual markers in interrelationships between MetS and inflammation is unknown.*
**What is New:** • *We uniquely demonstrate that within a multivariate model, waist circumference is the primary link between MetS variables and markers of inflammation in children.* • *Waist circumference may therefore be a useful population-level screening tool to identify future risk of CVD.*

## Introduction

Cardiovascular disease (CVD) continues to be a major cause of morbidity and premature mortality in developed countries and, to an increasing degree, in low- and middle-income countries as well. It has long been thought that CVD has its origins in childhood [21] and epidemiological evidence has revealed that many children exhibit at least one CVD risk factor [2, 30]. Type 2 diabetes mellitus (T2DM), which shares several risk factors with CVD, is now established as a condition not only affecting adults but also adolescents and children with resulting mortality and morbidity in early adult life [25]. The early identification in childhood or adolescence of those at risk of later CVD and T2DM may allow a targeted approach by lifestyle measures to prevent or delay the onset of these conditions in later life. Several of the risk factors for CVD and T2DM are related to obesity. Despite early indications that the prevalence of obesity in children has plateaued in several countries [22], childhood and adolescent obesity and their consequences are still major public health problems.

The prevalence of the metabolic syndrome (MetS), a cluster of cardiometabolic risk factors associated with an increased risk for the development of cardiovascular diseases and T2DM, is as high as 30–50% among overweight youth [17]. Traditional risk factors such as abdominal obesity, hypertension, elevated fasting blood glucose and triglycerides (TG), and reduced high-density lipoprotein cholesterol (HDL-C) are encompassed in the many definitions of the MetS. A joint scientific statement, led by the International Diabetes Federation and the American Heart Association along with four other bodies, defined MetS in adults as three abnormal findings out of the above five variables, with abdominal obesity indicated by waist circumference [1]. A consensus definition for MetS in children and adolescents has not yet been developed. There are currently at least 40 unique definitions of MetS for this age group [9], and the prevalence of MetS varies depending on the definition used [32]. Weiss and colleagues [33] argued that the environment plays a major role in the development of MetS, in particular the typical Western diet, which has now been adopted globally due to palatability and price. They suggest that MetS is instigated with mitochondrial overload and results in de novo lipogenesis, insulin resistance, and dysfunctional subcellular energy use.

Markers of systemic inflammation such as fibrinogen, C-reactive protein (CRP), interleukin-6 (lL-6), and adiponectin are also of considerable interest as potential predictors of the later development of CVD [6] and T2DM [34]. The evidence relating to CVD and T2DM risks, markers of inflammation and obesity in adolescents, has recently been reviewed [4, 13]. The adipocytes of visceral adipose tissue secrete a variety of cytokines and adipokines including interleukin-6 (IL-6) and, when hypertrophied, low levels of adiponectin. High concentrations of IL-6 and low concentrations of adiponectin are associated with systemic inflammation and insulin resistance, respectively. Systemic inflammation, which increases levels of CRP [7], stimulates pathological arterial intimal changes which are part of the basis for later CVD, while insulin resistance forms the key pathological change leading to T2DM. Even low levels of circulating CRP (2–6 mg/L), so called “high-sensitivity CRP” (hs-CRP), are indicative of an active inflammatory response to abdominal obesity in adults [26]. DeBoer [4] considered the evidence linking systemic inflammation to CVD risk in adults to be stronger than that relating to insulin resistance and T2DM but commented that long-term data linking increased hs-CRP levels—and increased insulin or decreased adiponectin—in childhood to adult disease outcomes are currently lacking. Nevertheless, he postulated that clinical and laboratory tests (among them, by implication, those relating to the MetS and inflammation as potential candidates) might be used as “screening tests” to facilitate the identification of adolescents at risk of CVD and T2DM with a view to ultimately improving prevention efforts.

Consensus is lacking regarding appropriate screening to identify the interrelated conditions of MetS and systemic inflammation in children which lead to the development of CVD in later life. Such advances require greater understanding of the separate contributions of each of the multiple components of MetS in the relationship between MetS and health outcomes, many of which are themselves multidimensional. In the previous studies of MetS, or cardiometabolic health, a univariate statistical approach is often used, which can mask the separate contributions of the MetS components. For example, it is common to classify MetS as a single condition (i.e., by classifying MetS as “present” or “absent”) [11] or by combining the components into a composite *z*-score [5]. Other studies that include the components of MetS conduct separate analyses for each of the components [15]. Multivariate statistical techniques have been used in the social sciences for many years, to investigate relationships between determinants (independent variables) and outcome variables (dependent variables) that are multidimensional in nature. These techniques are used less in the biological sciences, but are nevertheless valuable where the variables under investigation are multivariate in nature (i.e., they consist of several related dimension, as is the case with MetS). The purpose of this study was therefore to investigate the multivariate dimensionality and strength of the relationship between MetS components and inflammatory markers in adolescent children.

## Methods

The study is part of Prosiect Sir Gâr (The Carmarthenshire Project). Early results focusing on secular trends in CVD risk among 11–12 year olds have already been published [31]. The study was approved by the Dyfed-Powys NHS Research Ethics Committee.

### Participants

Pupils from three secondary schools in Carmarthenshire were asked to participate in the study which was integrated into the schools’ physical education program. Participant and parent/guardian information sheets and consent and assent forms were distributed to all year eight school children (age 12–14 years). Informed consent was obtained from all individual participants included in the study.

### Measures

All physical and physiological measurements were performed by the principal investigator and a trained research assistant. Protocols followed the Anthropometric Standardization Reference Manual [16]. Participants were dressed in shorts and t-shirt, and without footwear. Standing height was measured to the nearest millimeter using a portable stadiometer. Body mass was measured to the nearest tenth of a kilogram (kg) using a Phillips HP 5320 electronic scale (Philips UK, Ltd., Guilford, Surrey) calibrated against a balanced beam scale. Body mass index (BMI) was derived as the mass divided by stature squared (kg/m^2^) for each participant. Waist circumference was measured at the narrowest point between the lower rib border and the iliac crest using an anthropometric tape (Holtain Ltd., Crymych, Pembrokeshire, UK). Tanner stage maturational status was determined using a self-administered assessment form of secondary sexual characteristics that was previously validated against physical examination by a physician in 12–16-year-old children [29].

Blood pressure readings were taken using a Dinamap automatic BP monitor. Participants were seated, and blood pressure was taken in the morning with the participant fasted and not having undertaken vigorous exercise during the 30 min preceding the measurement. Blood pressure was recorded three times, and the average of the second and third reading was recorded.

Blood samples were collected between 9.00 am and 11.00 am, following an overnight fast. To control for plasma volume shifts, venous blood was sampled after the participant had assumed a seated position for at least 30 min [24]. Samples were analyzed for total cholesterol (TC), HDL-C, low-density lipoprotein cholesterol (LDL-C), TG, glucose, fibrinogen (Fg), high molecular weight (HMW) adiponectin, CRP, and IL-6. TC and TG concentrations were estimated by routine enzymatic techniques using the Vitros 950 System (Ortho-Clinical Diagnostics, Amersham, Bucks, UK). The concentration of HDL-C was determined after precipitation of very low-density and LDL-C with dextran sulfate and magnesium chloride. LDL-C concentration was calculated by the Friedewald formula [10]. Fibrinogen concentration was determined according to the method of Clauss [3] and using the ACL Futura analyzer (Instrumentation Laboratory Company, Lexington, MA). CRP concentration was measured by an immunoturbidimetric method using the Cobas Fara (Roche, Welwyn Garden, UK).

### Statistical analysis

All primary outcome variables were initially screened for outliers, data errors, and normality by running frequencies, calculating distribution statistics, and inspecting histograms. Three CRP scores reported as < 0.1 in the blood assay report were changed to 0.09, to facilitate statistical analysis. This pragmatic decision was taken on the basis that it would not meaningfully alter the distribution of the data and to exclude all data from participants who had contributed fully to the study would be otherwise unethical. Two high hs-CRP values were indicative of acute infection and were replaced using simple regression on IL-6, with which it had the highest bivariate correlation. Two variables (hs-CRP and IL-6) did not have a normal distribution, defined as having skewness and kurtosis values > |2.0|, and these were transformed using the natural log (Ln). This reduced skewness and kurtosis values below |2.0|, enabling their subsequent analysis using parametric statistics. Boys and girls were compared using independent *t* tests to determine any sex differences. Following significant differences for TG, glucose, waist circumference, adiponectin, LnCRP, and LnIL-6, sex-specific *z*-scores were calculated for these variables.

The primary research question was investigated using canonical correlation analysis. Canonical correlation analysis determines the multivariate association between two sets of variables, where each set consists of multiple related outcomes. In the current study, MetS comprises a set of related components, and inflammation was represented by a set of related inflammatory markers. Two canonical variates are calculated to obtain the maximal observed correlation between the two sets of variables (characterized here as the independent variables—diastolic blood pressure, systolic blood pressure, HDL-cholesterol, TG, glucose, and waist circumference, and the dependent variables—Fg, adiponectin, hs-CRP, and IL-6). Additional canonical variates are calculated to determine whether additional common variance can be explained. If significant canonical variates are found, canonical coefficients and correlations between the variables and their respective canonical variate help to explain the dimensionality of the relationship—specifically, which of the multiple variables on each side of the relationship is responsible for explaining the multivariate association. All statistical tests of significance were conducted at a nominal alpha level of *p* = 0.05.

There are no software packages nor reference tables for power associated with a canonical correlation analysis. However, the significance test for a canonical function is equivalent to a Pearson correlation coefficient (as it is the bivariate correlation between the two canonical variates representing the two sets of underlying variables, and each canonical variate is a single, composite value). Power analysis (G*Power version 3.1, Heinrich Heine Universität Düsseldorf) indicated that, in order to detect the frequently used ready-reference value of *r* = 0.30 (which equates approximately to 10% shared variance between the two sets of variables) at a power of 1-*β* = 0.80, a sample of *n* = 84 is required. A power of 1*-β* = 0.95 would be achieved with a sample of *n* = 138. Our sample therefore provided a high level of statistical power to detect any underlying correlations in the population.

## Results

Two hundred and sixty-eight participants took part in the study. All were of Caucasian origin. Table 1 shows the basic physical characteristics and biochemical measures of 229 participants for whom complete data for the canonical correlation analysis were available. Missing data were primarily for the blood-based variables and were due to either inability to collect blood, participant refusal, absence on the day of blood testing, or insufficient blood collected to conduct the full set of assays. Participants with missing blood data (*n* = 39) were compared to participants with complete blood data on age, BMI, sum of skinfolds, waist circumference, and both systolic and diastolic blood pressure. Generally, there were no significant differences between groups, except for age (mean difference = 0.1 year, *p* = 0.044) and systolic blood pressure (mean difference = 4 mmHg; *p* = 0.049). Maturational stage varied within the sample, although the majority were at Tanner stage 3 (39 and 35% based on breast/penis development and pubic hair distribution, respectively) or stage 4 (34%/41%).

**Table 1:** Physical characteristics and biochemical measures (mean ± SD) of participants

	Boys (*n* = 95)	Girls (*n* = 134)	All (*n* = 229)
Demographic variables			
Age (years)	13.54 (0.33)	13.49 (0.30)	13.51 (0.31)
Body mass (kg)	53.30 (13.23)	53.40 (11.44)	53.36 (12.18)
Height (m)	1.60 (0.90)	1.58 (0.66)	1.59 (0.77)
BMI (kg/m^2^)	20.60 (3.74)	21.31 (3.68)	21.02 (3.71)
Variables used in the multivariate analysis			
Fg (g/L)	2.69 (0.44)	2.65 (0.46)	2.66 (0.45)
HMW-Ad (ng/mL)	2484.20 (1605.01)	3381.78 (1798.25)	3009.42 (1773.36)
hs-CRP (mg/L)	0.67 (0.72)	0.44 (0.59)	0.54 (0.66)
IL-6 (ng/ml)	0.97 (1.09)	0.73 (0.56)	0.83 (0.83)
Diastolic BP	64.73 (11.10)	65.62 (10.19)	65.25 (10.56)
Systolic BP	116.59 (12.55)	115.78 (10.57)	116.11 (11.41)
HDL-C (mmol/L)	1.63 (0.33)	1.71 (0.32)	1.68 (0.33)
TG (mmol/L)	0.63 (0.29)	0.75 (0.30)	0.70 (0.30)
Glucose (mmol/L)	4.92 (0.35)	4.81 (0.27)	4.86 (0.31)
Waist circumference (cm)	68.41 (9.29)	65.79 (8.33)	66.88 (8.82)

*BMI* body mass index, *WC* waist circumference, *BP* blood pressure, *HDL-C* high-density lipoprotein cholesterol, *TG* triglyceride, *Fg* fibrinogen, *HMW-Ad* high molecular weight adiponectin, *hs-CRP* high-sensitivity C-reactive protein, *IL-6* interleukin-6

Pearson zero-order correlations within and between the two sets of variables are presented in Table 2. Within the set of four dependent variables, three of six correlations were significant at the *p* < 0.01 level, and within the set of six independent variables, 10 of 15 variables were significant, with three being significant at *p* < 0.05 and seven at the *p* < 0.01 level. The highest within-set correlations were between CRP and Fg (*r* = 0.58) and between diastolic and systolic blood pressure (*r* = 0.51). Of the 24 correlations between the independent and dependent variables, five were significant at the *p* < 0.05 level and nine were significant at the *p* < 0.01 level. The highest between-set correlations were between waist circumference and Fg (*r* = 0.51) and between waist circumference and CRP (*r* = 0.48).

**Table 2:** Zero-order correlations between and within dependent and independent variables

	Dependent variables				Independent variables					
	Fg	HMW-Ad (*z*)	LnCRP (*z*)	LnIL-6 (*z*)	DBP	SBP	HDL-C	TG (*z*)	Glucose (*z*)	WC (*z*)
Fg	1.0									
HMW-Ad (*z*)	− 0.10	1.0								
LnCRP (*z*)	0.58**	0.01	1.0							
LnIL-6 (*z*)	0.42**	0.02	0.41**	1.0						
DBP	0.28**	0.05	0.30**	0.24**	1.0					
SBP	0.12	− 0.03	0.14*	0.25**	0.51**	1.0				
HDL-C	− 0.21**	0.12	− 0.16*	− 0.17*	− 0.03	− 0.01	1.0			
TG (*z*)	0.28**	− 0.10	0.13*	0.09	0.20**	0.03	− 0.42**	1.0		
Glucose (*z*)	0.05	− 0.09	0.08	0.06	0.08	0.16*	− 0.07	0.16*	1.0	
WC (*z*)	0.51**	− 0.16*	0.48**	0.30**	0.33**	0.23**	− 0.33**	0.33**	0.13*	1.0

*Fg* fibrinogen, *CRP* C-reactive protein, *IL-6* interleukin-6, *BP* blood pressure, *HDL-C* high-density lipoprotein cholesterol, *TG* triglyceride, *(z)* sex-specific *z*-score

*p < 0.05; **p < 0.01

**Fig. 1:**
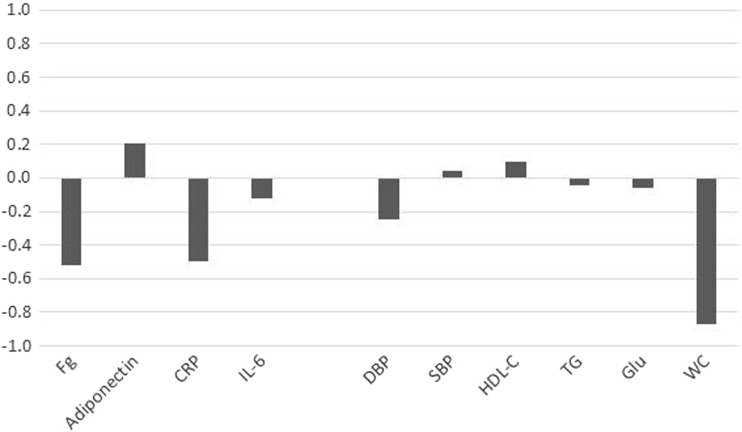
Canonical coefficients between MetS components and inflammation markers

The results of the canonical correlation analysis are shown in Tables 3 and 4. As shown in these tables, the pattern of results was similar regardless of whether the variables were adjusted for sex differences. We have focused on the sex-specific results, presented in Table 3, although the results were ostensibly the same. The pattern of coefficients is also illustrated in Fig. 1. The overall multivariate association between MetS and inflammation was significant (Wilks’ Lambda = 0.56, *p* < 0.001), indicating a significant association between the two sets of variables. There was 36% shared variance between the two sets of variables. Only the first canonical function was significant (*p* < 0.05). Inspection of the canonical coefficients (CC) in Table 3 helps to explain which of the underlying components of MetS and markers of inflammation are primarily responsible for the multivariate association. Similar to techniques such as factor analysis, the CCs can be viewed as loadings of each variable, or components, on the canonical variate. Higher CCs indicate a more statistically meaningful role played by the variable or components on the underlying canonical variate. Thus, Table 3 indicates that the multivariate relationship was explained primarily by the waist circumference component of MetS (CC = − 0.86) and inflammatory markers of fibrinogen (CC = − 0.58) and CRP (CC = − 0.44). CCs between HMW-adiponectin (CC = 0.20) and IL-6 (CC = − 0.11) and the inflammation canonical variate were much lower.

**Table 3:** Canonical correlation analysis results for sex-specific *z*-scores

	Standardized canonical coefficient	Correlation between variable and canonical variate
Dependent variables		
Fg	− 0.58	− 0.90
HMW-adiponectin (*z*)	0.20	0.25
LnCRP (*z*)	− 0.44	− 0.82
LnIL-6 (*z*)	− 0.11	− 0.54
Independent variables		
Diastolic BP	− 0.25	− 0.52
Systolic BP	0.03	− 0.28
HDL-C	0.06	0.38
TG (*z*)	− 0.04	− 0.39
Glucose (*z*)	− 0.02	− 0.14
Waist circumference (*z*)	− 0.86	− 0.97

*Fg* fibrinogen, *CRP* C-reactive protein, *IL-6* interleukin-6, *BP* blood pressure, *HDL-C* high-density lipoprotein cholesterol, *TG* triglyceride, *(z)* sex-specific *z*-score

**Table 4:** Canonical correlation analysis results for non sex-specific *z*-scores

	Standardized canonical coefficient	Correlation between variable and canonical variate
Dependent variables		
Fg	− 0.52	− 0.87
HMW-adiponectin (*z*)	0.21	0.29
LnCRP (*z*)	− 0.50	− 0.85
LnIL-6 (*z*)	− 0.12	− 0.55
Independent variables		
Diastolic BP	− 0.25	− 0.50
Systolic BP	0.04	− 0.28
HDL-C	0.10	0.41
TG (*z*)	− 0.04	− 0.30
Glucose (*z*)	− 0.06	− 0.20
Waist circumference (*z*)	− 0.87	− 0.97

*Fg* fibrinogen, *CRP* C-reactive protein, *IL-6* interleukin-6, *BP* blood pressure, *HDL-C* high-density lipoprotein cholesterol, *TG* triglyceride, *(z)* sex-specific *z*-score

## Discussion

This study presents a novel approach to understanding the link between MetS and inflammatory markers in a large sample of apparently healthy school children. Rather than the traditional method of using MetS variables to classify children’s metabolic status as MetS present/absent, we instead investigated all MetS variables as separate, but interrelated, continuously distributed components. Metabolic status is a multiple variable or multidimensional construct. Although evidence-based criteria have been developed to classify MetS status as present/absent, there has been inconsistency in the literature regarding what definitions should be used [14]. A review of the topic identified 40 unique definitions of MetS in children and adolescents [9]. While an attempt at harmonizing the definition for adults has been made jointly by several bodies [1], a consensus definition for children and adolescents is lacking. Dichotomous variable categorizations are difficult to define in a clear and unbiased way due to lack of specific normative values for all criteria included in the syndrome and for all populations [17]. Further, use of the MetS concept with children remains controversial [23]. Indeed, it has been suggested that as the application of the MetS model has not been fully validated in children and adolescents as yet, diagnosis, prevention, and treatment should better focus on established risk factors rather than the diagnosis of MetS [14]. Finally, classification of MetS as a univariate, dichotomous construct does not enable investigation of the contribution of individual components to important physiological mechanistic variables such as inflammatory markers. The same is true of expressing metabolic status as a composite score (by combining the *z*-scores for separate components), which (a) results in a single score and (b) weights every component equally, potentially masking a differential contribution to health risk of each of the components. The multivariate analysis used in this study demonstrates the importance of allowing the MetS components to be investigated simultaneously within a single analysis, while also enabling the teasing out of separate contributions of each of the underlying components.

Results of the canonical correlation analysis point clearly to waist circumference being the component of the MetS most strongly associated with markers of systemic inflammation. Of the plasma markers of inflammation, fibrinogen and CRP primarily explain this association. Thus, waist circumference, as a surrogate for central adiposity, was the strongest predictor of the inflammatory aspect of CVD risk in these Caucasian adolescents. While a relationship between waist circumference and inflammation has previously been demonstrated in cross-sectional studies [28], we establish for the first time the multivariate dimensionality and strength of this relationship considering other components of the MetS. This finding lends support for the inclusion of abdominal obesity, indicated by a waist circumference greater than the relevant age-specific cutoff point, as an obligatory component in the definition of metabolic syndrome in children and adolescents by the International Diabetes Federation [35]. The mandatory use of adiposity to define MetS has been subject to much debate (for example see Zivkovic and German (2007) [36]). This paper, therefore, will add to the evidence base should attempts be made to reach a consensus definition, as has already been achieved for adults [1].

While, to our knowledge, this is the first study that has investigated associations between MetS and inflammation in this age group, using a multivariate approach, some similarities existed within our univariate analyses with other large-scale studies of MetS in children and youths. In a study of girls and boys aged 6–14 years [18], a matrix of 153 zero-order correlations was reported between a set of cardiometabolic risk variables and anthropometric variables. The ratio of waist/height correlated with a set of eight lipid markers, fasting glucose, and resting blood pressure, with an average correlation of *r* = 0.14 across the eight variables. In our study, waist circumference correlated with the set of five lipid markers, fasting glucose, and resting blood pressure variables with an average Pearson correlation of *r* = 0.27. Waist/height ratio has also been reported as being correlated with systolic blood pressure (*r* = 0.25) [19] and triglyceride/HDL-C ratio (*r* = 0.25) [20] in two other studies of children and adolescents between 6 and 14 years of age. Waist circumference variables have therefore been shown to correlate with other MetS variables in samples of children and youths previously, albeit these studies did not report any markers of inflammation. Interestingly, in our study, waist circumference correlated highly and significantly with three of the four inflammatory markers, and generally correlated more highly with the inflammatory markers than with the other MetS components.

These findings regarding waist circumference may also have practical implications for possible steps in diagnosis of and screening for metabolic syndrome at individual and population level. Any consideration of clinical and biochemical parameters for the early identification of CVD and T2DM risk in children and adolescents must consider not only what parameters would be most useful and feasible to measure, but also what is meant by “screening” as a means of early detection. Sackett and Holland (1975) drew important distinctions between the detection of disease in the context of diagnosis (in which individual patients relate to individual clinicians) and “case-finding” (screening as a population-based procedure) [27]. Population-based screening, in which apparently healthy individuals are invited to participate, has the implicit promise of benefits over and above those which would accrue if individuals had waited until they sought medical advice as individuals. For early detection of risk of CVD and T2DM, any advocate of population-based screening must be assured that there is adequate evidence of the benefits and accuracy of early detection by screening. While acknowledging that the cross-sectional nature of our findings limits the ability to infer that current associations would be effective in predicting long-term risk, and noting the moderately sized, homogenous sample recruited, the data presented here suggest an approach for screening. Measurement of waist circumference together with the laboratory measurement of fibrinogen and CRP could be considered as possible candidates for a screening program. Waist circumference is non-invasive and low-tech and can be carried out by a professional—easily trained in the correct technique—as part of the clinical consultation with an individual patient or as part of population-based activities such as school-based health programs. Venous blood sampling for fibrinogen and CRP measurement is somewhat more invasive and requires laboratory facilities. In a population-based program, a two-stage process might be envisaged—first waist circumference and then, on a high-risk sub-set, fibrinogen and CRP measurement. Preference for selective screening of high-risk subgroups of the population has long been advocated [8].

### Limitations and strengths

Generalization of these results to other populations is limited by the sample. Based on our sample, generalization should therefore be restricted to Caucasian youths between the ages of 12 and 14 years. Additionally, although maturational status was related to MetS in previous studies [32], we were unable to investigate this in our study due to the relative homogeneity of maturational status in our sample (approximately 75% of the sample were at Tanner stages 3 and 4). Strengths of the study include use of a novel and appropriate analysis technique, canonical correlation analysis that allowed the MetS components to be investigated simultaneously within a single analysis. This method was previously used to examine the relationships between components of physical activity and cardiorespiratory fitness versus CVD risk factors in children and adolescents [12].

## Conclusions

We demonstrate for the first time that within a multivariate model, waist circumference is the primary link between MetS variables and markers of inflammation in Caucasian school children. We suggest that this measure of central adiposity may be a useful population-level screening tool to identify future risk of CVD. Future research into MetS and CVD risk factors should consider multivariate statistical approaches, in order to identify the separate contributions of each dimension in interrelationships and to identify which dimensions are most influenced by preventive interventions.

## Abbreviations

CC: Canonical coefficient
CRP: C-reactive protein
CVD: Cardiovascular disease
Fg: Fibrinogen
HDL-C: High-density lipoprotein cholesterol
HMW: High molecular weight
IL-6: Interleukin-6
LDL-C: Low-density lipoprotein cholesterol
MetS: Metabolic syndrome
T2DM: Type 2 diabetes mellitus
TC: Total cholesterol
TG: Triglycerides
